# Editorial: Organoids, organs-on-chip, nanoparticles and *in silico* approaches to dissect the tumor-immune dynamics and to unveil the drug resistance mechanisms to therapy in the tumor microenvironment

**DOI:** 10.3389/fimmu.2023.1253551

**Published:** 2023-07-18

**Authors:** Fabrizio Mattei, Jason T. George, Mohit Kumar Jolly

**Affiliations:** ^1^ Department of Oncology and Molecular Medicine, Istituto Superiore di Sanità, Rome, Italy; ^2^ Department of Biomedical Engineering, Texas A&M University, College Station, TX, United States; ^3^ Center for BioSystems Science and Engineering, Indian Institute of Science, Bangalore, India

**Keywords:** preclinical models, advanced *in vitro* models, 3D models, advanced algorithms for immuno-oncology, personalized medicine for cancer patients, organs-on-chip platforms, nanotechnologies, drug delivery

The relationships between cancer and immune system are under intense investigation and have currently made giant steps thanks to the great development of advanced *in vitro* models as useful preclinical systems (1–4). Advanced models of study represent a large set of tools that are important for enabling scientists to study in detail specific biological events, including those related to the multifaceted dynamics of the tumor microenvironment (TME) and the associated phenomena such as drug resistance and crosstalk between cancer and immune cells.

In this Research Topic we collected a set of key articles (6 Research papers and 6 Review articles) exploring diverse aspects of the TME. Advanced models of study, such as *in vitro* 3D models, Organs-on-Chip (OOC), and mathematical algorithms for onco-immunology applications, including multi-omics models, have been discussed in these articles.

In their Research article, Brummer et al. proposed a quantitative advanced mathematical model based on Sparse Identification of Non-linear Dynamics (SINDy) algorithm (5), applied to a real biological system in order to discover cell-cell interaction dynamics in *in vitro* experimental data. They employed such a model to specifically investigate the interaction dynamics of CAR T-cell populations and glioblastoma. In the future, this model may be applied to optimize the efficacy of CAR-T-based therapies in aggressive and inoperable tumors such as glioblastoma.

Carannante et al. contributed a Review on how *in vitro* 3D systems can be further implemented to reach high-fidelity recapitulation of the tumor microenvironment to study the activity of Natural Killer (NK) cells. NK cells are key actors participating in cancer immunosurveillance and maintaining tissue homeostasis (6). The Authors proposed multiple ways to implement the existing 3D platforms to develop spheroids and organoids, useful to generate specific systems allowing researchers to monitor in real time the NK behavior (i.e., Caspase activity within spheroids, quantitative evaluation of NK infiltration into spheroids) and NK-based therapeutic efficacy.

Foxall et al. developed a 3D spheroid model for real time monitoring of antibody-specific therapy in diffuse large cell B cell lymphoma (DLBCL). This neoplastic disease is one of the most common types of non-Hodgkin lymphoma (7). This advanced 3D model was shown to be a reliable tool to investigate how cancer-associated fibroblasts (CAFs) and tumor-associated macrophages (TAMs), important constituents of the TME, interact with DLBCL. This model accurately recapitulates key features of the DLBCL TME necessary for elucidating TAM-CAF direct and indirect crosstalk.

Pancreatic ductal adenocarcinoma (PDAC) is an aggressive type of cancer (8) for which Geyer et al. evaluated the impact of the tumor stromal components on immune cell distribution and recruitment in a PDAC advanced preclinical model. The dense stromal microenvironment of PDAC creates a barrier for immune cell infiltration and poses a challenge for immunotherapeutic strategies. The study establishes a 3D PDAC model cultured under flow, consisting of an endothelial channel, pancreatic stellate cells (PSCs), and PDAC organoids, to investigate the role of the TME on immune cell recruitment. The findings suggest that stromal cells form a physical barrier and a biochemical microenvironment that influences immune cell distribution. Additionally, targeting the stroma led to an increase in immune cell infiltration. This study highlights the potential of this model for contributing to understanding the cellular interactions and identifying key players in the immunosuppressive moiety of PDAC.

In their Research article, Huang et al. exploited a multi-omics approach to evaluate transcriptional and epigenetic regulation of GPRC5B, a G protein-coupled receptor (GPCR) (9) associated to macrophages activity. In their proposed pipeline, the Authors used a model employing RNA sequencing (RNA-seq) assay for transposase-accessible chromatin using sequencing (ATAC-seq) and chromatin immunoprecipitation with high-throughput sequencing (ChIP-seq). They were able to show that GPRC5B represent a central GPCR for colon adenocarcinoma prognosis by direct modulation of the transcription factor (TF) GATA4. This study is a representative example on the usefulness of multi-omics approaches for TF investigations to optimize cancer therapy.

Xie et al. showed a Research article on kidney renal clear cell carcinoma (KIRC), one of the most lethal tumors of the urinary tract, with limited treatment solutions and poor prognosis (10). By using the R Limma package, the Authors exploited a multi-omics approach to find associations between prognosis and epigenetic modifications in KIRC patients. Of note, they identified a set (8 genes) of dysregulated epigenetic protein coding genes (epi-PCGs) (11) that can be successfully employed to evaluate KIRC prognosis. Specifically, such a signature predicted a strong association with KIRC prognosis. Indeed, patients with a high signature score experienced significantly worse clinical trajectories than those with a low score, thus suggesting that the use of a restricted epi-PCG gene set could provide a useful prognostic signature to investigate on pathological mechanisms of KIRC. This is an important prerequisite for developing novel and efficacious KIRC-specific drug targets.

In their Perspective article, Kumar et al. debated the value of commonly used response grading criteria in early oncology trials, namely the Response Evaluation Criteria In Solid Tumors (RECIST), version 1.1 (v1.1) (12). The Authors argue that RECISTv1.1 is ambiguous regarding lesion-to-lesion variation and can introduce bias in decision-making problems. They provided theoretical examples of how lesion-to-lesion variability can lead to misclassification of patient response. The Authors review immune checkpoint inhibitor (ICI) clinical trial data and find that lesion-to-lesion heterogeneity is widespread in ICI-treated patients. They then conclude that the incorporation of lesion-to-lesion heterogeneity through Quantitative Systems Pharmacology (QSP) models can constitute a complementary implementation to the RECISTv1.1 model. Therefore, ameliorating decision-making processes in early-stage oncology drug development can lead to key benefits in cancer patient care.

Manduca et al. contributed a Review article overviewing the current advanced *in vitro* models utilized to investigate on the interactions between immune and cancer cells to better recapitulate the complexity of the TME dynamics (3, 13–15). The Authors also discussed advantages and disadvantages of employing complex *in vitro* systems such as spheroids, organoids and organs-on-chip platforms (OOC). Accessibility versus fidelity represent two central parameters to appropriately chose the ideal platform to study a specific TME event. Simultaneous control of many relevant biological factors facilitates the use of accessible systems, such as spheroids or organoids, whereas if one intends to reproduce an event occurring in the TME, the use of OOC platforms is highly beneficial due to its high-fidelity recapitulation of the TME events. This article highlights that advanced *in vitro* systems are invaluable tools for studying the TME, considering that it is both very difficult and expensive to analogously do so *in vivo*.

Shen et al. proposed a review article overviewing the impact of the nanomaterials and the associated bioprinting methods in the use of organoids and spheroids. Nanomaterials can represent a very useful architectural implementation when generating organoids or spheroids in 3D culture systems, which can assist to better maintain the unit stability, affordability, and recapitulation fidelity (16). For example, a new methodology based on the use of neodymium magnets (17) is debated, which allows spheroids to be developed under the action of magnetic fields. Nanomaterials are also used to improve the efficiency of drug delivery and screening in spheroids and organoid units. Therefore, using specific types of nanomaterials can be very useful for the development of high-fidelity and highly accessible spheroid/organoid units, which can be properly optimized to recapitulate specific events of the TME.

In a Review article, Sun et al. provided an exhaustive overview of the various culture methods for tumor organoids (18) to study the complexity of the TME and to emulate their associated dynamics. Specifically, they debated the pros and cons of three main culture techniques, namely submerged Matrigel culture, air-liquid interface culture, and microfluidic 3D culture. They also discussed on how these different organoid culture methods can be applied to evaluate immunotherapeutic strategies, such as adoptive cell transfer, ICI and other antibody-based therapies. Collectively, these approaches represent promising tools for personalized medicine in cancer.

Warwas et al. proposed a Research article in which the use of co-stimulatory bispecific antibodies (BiMAb) (19) in breast cancer models has been addressed to enhance T cell activation and tumor cell killing. The study analyzed various BiMAb targeting breast cancer antigens and bi-functional fusion proteins targeting tumor necrosis factor ligand (TNFL) superfamily members. The functional activity of the BiMAb was assessed using tumor cell lines and purified T cells in monolayer and tumor spheroid models. The results showed that the combination treatment of BiMAb with co-stimulatory antibodies significantly enhanced T cell activation, proliferation, cytokine secretion, and tumor cytotoxicity. Co-stimulation also overcame the immunosuppressive effects of TGF-β and IL-10. The study suggests that co-stimulatory BiMAb, assayed in advanced tumor models of study (spheroids), could provide a more localized and effective activation of the immune system in breast cancer treatment.

In their Review article, Yoon et al. discussed the advancements made in the culture methods for colorectal cancer (CRC). CRC currently represents one of the most widespread type of solid malignancy (20). Here, the pros and cons associated to 2D, 3D, and complex preclinical models (including OOCs), have been reported in detail for CRC in order to reflect on the optimal way to recapitulate and investigate the CRC TME. Their rational and optimized use (i.e., exact type of cells and hydrogel to be loaded in the OOC; defined microstructure of the OOC) constitutes a crucial step in studying the immune microenvironment of CRC and evaluating the effectiveness of immunotherapies.

In conclusion, this Editorial represents a relevant reference to those researchers specifically interested in developing novel ideas based on the use of new preclinical advanced models (such as organoids and OOCs), algorithms based on multi-omics approaches and drug delivery nanotechnologies to be applied in immuno-oncology. These systems represent promising biotools for TME mimicking studies and for the optimization of cancer therapy strategies in the context of cancer patient’s personalized medicine.

## Author contributions

All authors listed have made a substantial, direct, and intellectual contribution to the work and approved it for publication.
